# Plakoglobin expression in fibroblasts and its role in idiopathic pulmonary fibrosis

**DOI:** 10.1186/s12890-015-0137-5

**Published:** 2015-11-06

**Authors:** Stephanie A. Matthes, Thomas J. LaRouere, Jeffrey C. Horowitz, Eric S. White

**Affiliations:** Division of Pulmonary and Critical Care Medicine, Department of Internal Medicine, University of Michigan Medical School, Ann Arbor, MI 48109-5642 USA

**Keywords:** Idiopathic pulmonary fibrosis, Fibroblasts, Plakoglobin, Fibronectin

## Abstract

**Background:**

Idiopathic pulmonary fibrosis (IPF) is an interstitial fibrotic lung disease of unknown origin and without effective therapy characterized by deposition of extracellular matrix by activated fibroblasts in the lung. Fibroblast activation in IPF is associated with Wnt/β-catenin signaling, but little is known about the role of the β-catenin-homologous desmosomal protein, plakoglobin (PG), in IPF. The objective of this study was to assess the functional role of PG in human lung fibroblasts in IPF.

**Methods:**

Human lung fibroblasts from normal or IPF patients were transfected with siRNA targeting PG and used to assess cellular adhesion to a fibronectin substrate, apoptosis and proliferation. Statistical analysis was performed using Student’s t-test with Mann–Whitney post-hoc analyses and results were considered significant when *p* < 0.05.

**Results:**

We found that IPF lung fibroblasts expressed less PG protein than control fibroblasts, but that characteristic fibroblast phenotypes (adhesion, proliferation, and apoptosis) were not controlled by PG expression. Consistent with this, normal fibroblasts in which PG was silenced displayed no change in functional phenotype.

**Conclusions:**

We conclude that diminished PG levels in IPF lung fibroblasts do not directly affect certain phenotypic behaviors. Further study is needed to identify the functional consequences of decreased PG in these cells.

## Background

Idiopathic pulmonary fibrosis (IPF) is a chronic progressive fibrotic disorder of the lung with no clear etiology [1] and without proven therapies to impact mortality. Alveolar epithelial cell injury may be an initiating factor in fibrotic lung repair, which might reflect an aberrant recapitulation of developmental programs as a means to repair the injured tissue [2]. With this in mind, recent work has begun to evaluate the role of cellular adhesion proteins as mediators responsible for the progression of IPF [3], with both junctional and non-junctional properties being investigated. As an example, the Wnt pathway is typically activated during development, but is thought to become re-activated in IPF as an epithelial repair response [3–7]. Experimental evidence suggests that blockade of this pathway may in fact attenuate the fibrotic response in experimental models [8–10].

Desmosomes are intercellular junctions that provide adhesion and tensile strength to tissues by anchoring intermediate filaments to the cell surface [11, 12]. Desmosomes are instrumental in providing and maintaining stability to tissues under high mechanical strain while also serving as orchestrators of signaling molecules. Plakoglobin is an Armadillo-repeat protein that is highly homologous to β-catenin and functions as a junctional protein in the desmosome. In light of its homology with β-catenin, PG has previously been shown to regulate Wnt signaling [13, 14]. However, it is also clear that PG plays a so-called ‘non-junctional’ role other than providing intercellular adhesion and strength to opposing cells. Despite evidence suggesting that non-junctional roles of PG may be important in regulating cell biology, no studies to date have examined these properties in fibrotic pulmonary mesenchymal cells.

One of the hallmarks of IPF is injury to the alveolar epithelial cells and subsequent recruitment of fibroblasts and myofibroblasts. Myofibroblasts are differentiated fibroblasts that express α-smooth muscle actin in organized stress fibers that confer the differentiated cell with contractile properties similar to smooth muscle. This contraction, along with the synthesis of extracellular matrix, positions the myofibroblast as a critical effector cell in the wound-repair response [15]. Neighboring myofibroblasts communicate through mechanical adhesion structures such as the adherens junction (AJ), which contain single-pass transmembrane cadherins, p120-catenin, β-catenin, and PG proteins that bind to α-actinin and subsequently actin filaments [16].

Fibronectin (Fn) is a fibrillar multimeric protein that serves as an integral linker protein which strengthens and stabilizes the extracellular matrix [17]. Cross-talk between cell-cell and cell-matrix adhesion, in particular between PG and Fn have been shown in PG^−/−^ keratinocytes where cell motility, focal adhesions and Fn (*Fn1*) mRNA stability were altered [18].

To determine whether PG affects the physiological behavior of pulmonary fibroblasts, we examined the adhesive properties of fibroblasts to a cellular fibronectin substrate as well as the influence of PG expression on proliferation and apoptosis.

## Methods

### Cell culture

Primary lung fibroblasts were explanted from human distal lung as previously described [19]. Normal fibroblasts were isolated from organ donors whose lungs were deemed unsuitable for transplantation. IPF fibroblasts were isolated from lungs of patients at time of transplantation. Lung samples were obtained from Gift of Life Michigan and the use of tissues was pre-approved by Gift of Life Michigan. The University of Michigan Institutional Review Board has reviewed this protocol and deemed it exempt from oversight as all samples were completely de-identified prior to acquisition. All cells were used between passages 2 and 9, and were maintained in Dulbecco’s modified eagle’s medium (DMEM) supplemented with 10 % fetal bovine serum, 10 mM HEPES, 100 U/mL penicillin, 100 U/ml streptomycin, and 2.5 mg/L Amphotericin B.

### siRNA-mediated knockdown of plakoglobin (PG)

PG was silenced in primary lung fibroblasts using stealth RNAi™ from Invitrogen (Life Technologies, Grand Island, NY). The siRNA-PG sequence was: 5′-UCAAGUCGGCCAUUGUGCAUCUCAU-3′. The non-targeting control construct (ΦsiRNA-PG) used in all control experiments contained the following sequence: 5′-AUGCUGAGUACACUAACGGAGCUGA-3′. Transfection was carried out using Lipofectamine RNAi™Max (Invitrogen) and serum free media. After 24 hours, media with 3 % fetal bovine serum was added and used in the experiments described below 72 hours post-transfection.

### Semi-Quantitative Real Time Polymerase Chain Reaction (SQ RT-PCR)

SQ RT-PCR was used to quantify endogenous levels of plakoglobin in whole lung tissue (*jup*: Fwd: CCGAGGACAAGAACCCAGAC, REV: GTGGCATCCATGTCATCTCC) and GAPDH (FWD: GTC TTC ACT ACC ATG GAG AAG G, Rev: TCATGGATGACCTTGGCCAG) as previously described [20, 21]. Primers were designed using the NCBI/Primer-Blast tool and the comparative cycle threshold approach was used to determine relative gene expression patterns [22]. The relative expression level of the gene (Δ*C*t) was calculated as follows: *C*t _JUP_-*C*t _GAPDH_, and the relative expression level of *jup* was calculated using the 2^-ΔΔ*C*t^ method.

### Western blots

Western blots were performed as previously described [19, 21]. Primary antibodies include GAPDH (Cell Signaling Technology, 1:1000), poly ADP ribose polymerase (PARP) (Cell Signaling Technology, 1:1000) and PG (BD Transduction Laboratories, 0.25 μg/mL). Secondary antibodies (ThermoFisher Scientific) included anti-mouse IgG HRP (for PG) or anti-rabbit IgG HRP (for GAPDH and PARP). GAPDH was used as a loading control for all primary antibodies. Bands were developed using SuperSignal® West Pico Chemiluminescent Substrate (ThermoFisher Scientific).

### Adhesion assays

A 96-well assay plate (Corning Incorporated, Costar®) was coated with either cellular fibronectin (cFN) at 20 μg/mL or left uncoated for the bovine serum albumin (BSA) control and incubated overnight at 4 °C. Plates were subsequently washed with PBS to remove unbound cFN and blocked for 30 minutes at 37 °C with 1 % BSA in serum-free DMEM. Cells were rinsed with Hanks’ Balanced Salt Solution (HBSS) without magnesium or calcium chloride (Gibco, Life Technologies) and harvested using 2 mM EDTA. Following detachment, HBSS containing both magnesium and calcium chloride was added in equal volume to the EDTA solution. Cells were centrifuged at 318 × *g* and the pellet was re-suspended in serum-free DMEM. Cells were counted, seeded at a density of 50,000 cells/well, then centrifuged at 8 × *g* and incubated at 37 °C and 5 % CO_2_ for one hour. Plates were then centrifuged inverted for 5 minutes at 50 × *g* to remove unattached cells. Remaining cells were fixed at room temperature with 0.5 crystal violet, 1 formaldehyde and 20 % methanol in double-distilled water. The plate was rinsed with PBS and fluorescence emission was measured in a microplate reader (SpectraMax M5 microplate reader, Molecular Devices, Sunnyvale, CA) at 595 nm.

### Apoptosis assay

Apoptosis was induced in serum-starved fibroblasts by treating with anti-FasL antibody (human, activating; Millipore (Billerica, MA), 0.5 μL/mL) alone or in conjunction with cycloheximide (Sigma-Aldrich, 1 μL/mL) for 6 hours or overnight. Cell lysates were harvested and assayed for cleavage of PARP by immunoblot as described [23] or processed for flow cytometry using Annexin V and propidium iodide (Alexa Fluor® 488 Annexin V/Dead Cell Apoptosis Kit, Life Technologies). Samples prepared for flow cytometry according to manufacturer’s protocol were immediately processed on a MoFlow Astrios™ cell sorter (Beckman Coulter, Brea, CA) [24].

### Proliferation assays

Dishes were coated with cell-derived fibronectin (purified from conditioned media of human lung fibroblasts; 20 μg/ml) then rinsed and blocked as described above. Cells were seeded at 5000 cells/well in triplicate wells per condition and incubated with 1 % serum + DMEM in addition to 20 ng/mL of Platelet Derived Growth Factor (PDGF, R&D systems, Minneapolis, MN) or 2 ng/mL of human recombinant TGF-β1 (R&D systems, Minneapolis, MN). The cells were then incubated at 37 °C and 5 % CO_2_ for 24 hours. Proliferation was assessed using the CyQuant® NF Cell Proliferation assay (Life Technologies) following the manufacturer’s instructions. Fluorescence intensity was measured with a microplate reader. Blank wells containing dye only and background (cells, no dye) were subtracted from the average emission of three wells for each condition.

### Statistical analysis

All experiments were analyzed using GraphPad Prism 6.02 (La Jolla, CA). Results were considered significant when *p* < 0.05 using the nonparametric Mann–Whitney U test. All results were reported using the mean ± SEM.

## Results

### Plakoglobin expression in normal and IPF lung fibroblasts

**Fig. 1:**
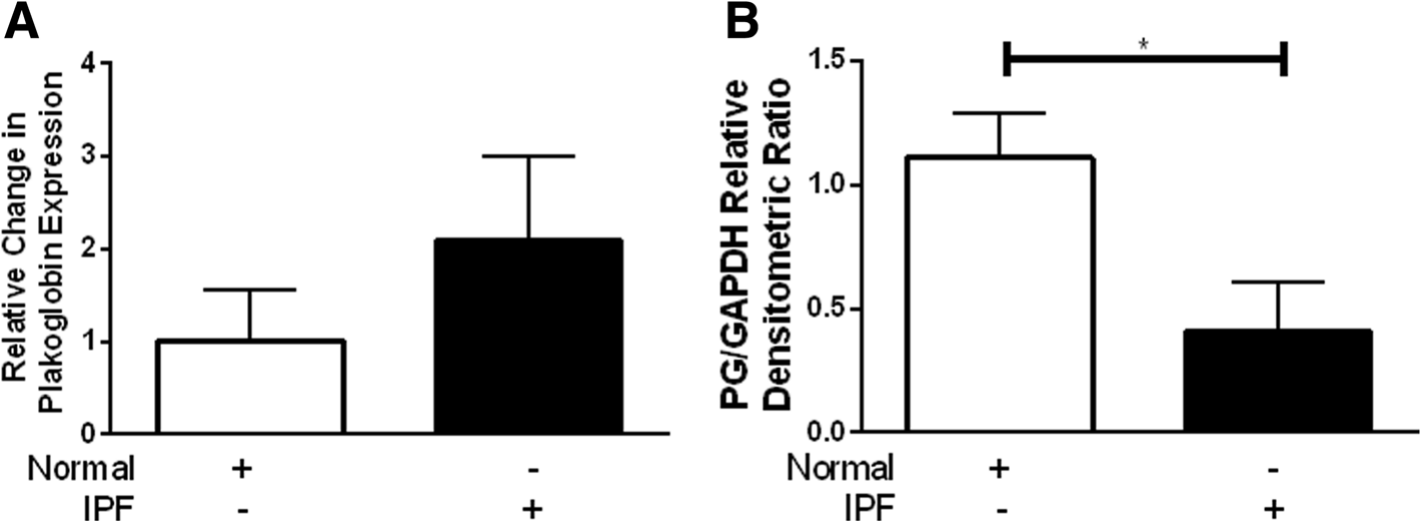
Plakoglobin (PG) RNA and protein expression levels between normal and IPF whole lung. Each normal (*white*, *n* = 7) or IPF (*black*, *n* = 8) whole lung lysate was assessed for PG RNA expression by RT-PCR and shown in a grouped based on classification. Relative change in PG (*jup*) expression was obtained by comparing PG to GAPDH. PG transcript levels in IPF lungs showed a trend toward increased expression, but were not statistically significantly different from normal fibroblasts (**a**). Normal (*white*, *n* = 6) or IPF (*black*, *n* = 5) whole lung lysates were assessed for PG protein expression by western blot. There is a significant decrease in PG protein expression in IPF lung tissue compared to the normal lung tissue (**b**, *p* = 0.04)

**Fig. 2:**
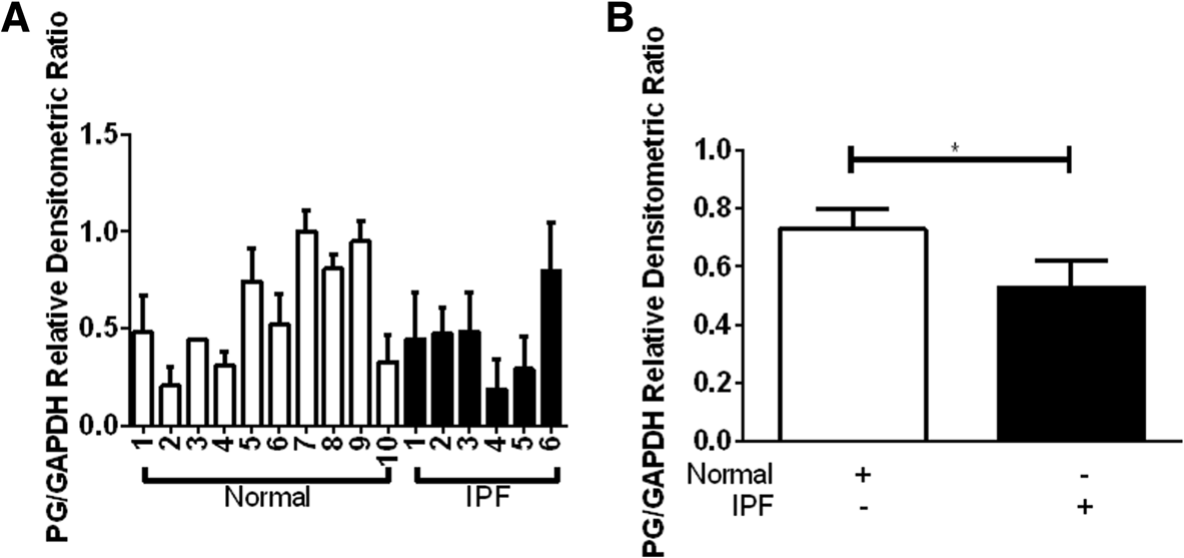
IPF pulmonary fibroblasts have reduced level of PG protein expression. Each normal (*white*) or IPF (*black*) cell line was assessed for PG protein expression by Western blot and shown as individual protein expression in (**a**) or grouped based on classification (**b**). The individual cell line levels (*left panel*) are normalized to S127N. Densitometric ratios were obtained by comparing PG to GAPDH. **b** When the cells are grouped the IPF cells overall show a significant decrease in PG expression compared to normal cells (Normal, *n* = 46 and IPF, *n* = 31, *p* < 0.05)

Because of the potential role for plakoglobin in mediating Wnt/β-catenin activity, we first sought to evaluate the RNA and protein expression of plakoglobin in primary lung fibroblasts from normal controls or IPF patients. The RNA expression profile for plakoglobin was not significantly different between normal (*n* = 7) and IPF patients (*n* = 8) (Fig. 1); however protein expression levels of plakoglobin did appear to be significantly lower in IPF whole lung homogenates (*n* = 5) compared to normal whole lung homogenates (*n* = 6), suggesting possible increased turnover or decreased protein translation. PG protein levels in explanted fibroblasts demonstrated heterogeneity within both the normal and IPF populations (Fig. 2a). Despite this heterogeneity, densitometric analysis revealed a small but statistically significant decrease in plakoglobin expression from the IPF fibroblast population in comparison to the control cells (Fig. 2b, Normal, *n* = 46 and IPF, *n* = 31, *p* = 0.05).

### Loss of Plakoglobin does not impact fibroblast adhesion, proliferation, or apoptosis

**Fig. 3:**
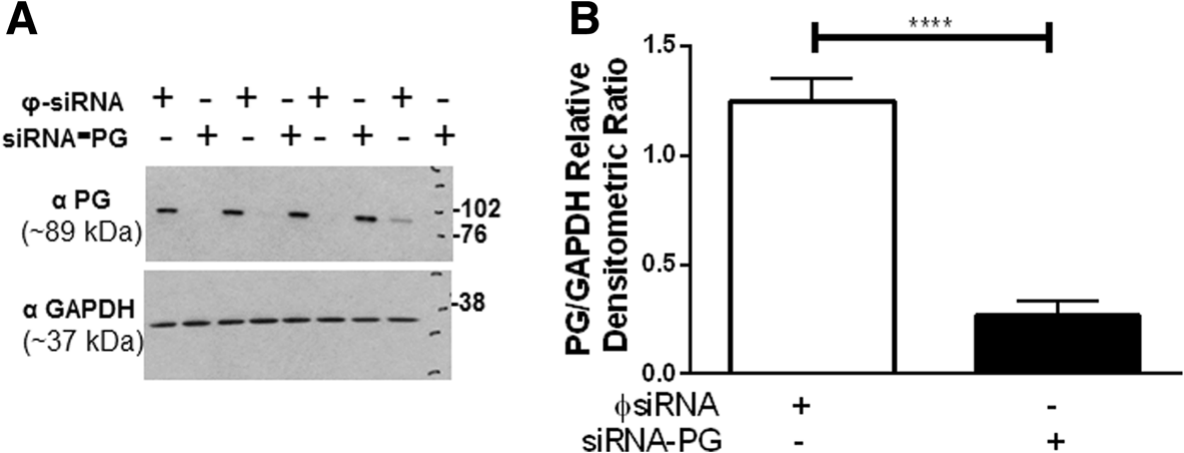
Successful knockdown of PG in lung fibroblasts. Treatment of lung fibroblasts with siRNA-PG dramatically reduces cellular PG protein expression as shown in a western blot (**a**). On average the cells treated with siRNA-PG achieved a significant reduction ~79 % efficient knockdown of PG protein (****p < .0001) (**b**)

To determine whether a reduction in plakoglobin was associated with functional consequences, we used siRNA to silence it using in primary normal lung fibroblasts. Figure 3, demonstrates the near-complete knockdown of plakoglobin in cells transfected with the specific siRNA (siRNA-PG). As expected the control siRNA (φ-siRNA) had no significant impact on PG expression in these normal lung fibroblasts.

**Fig. 4:**
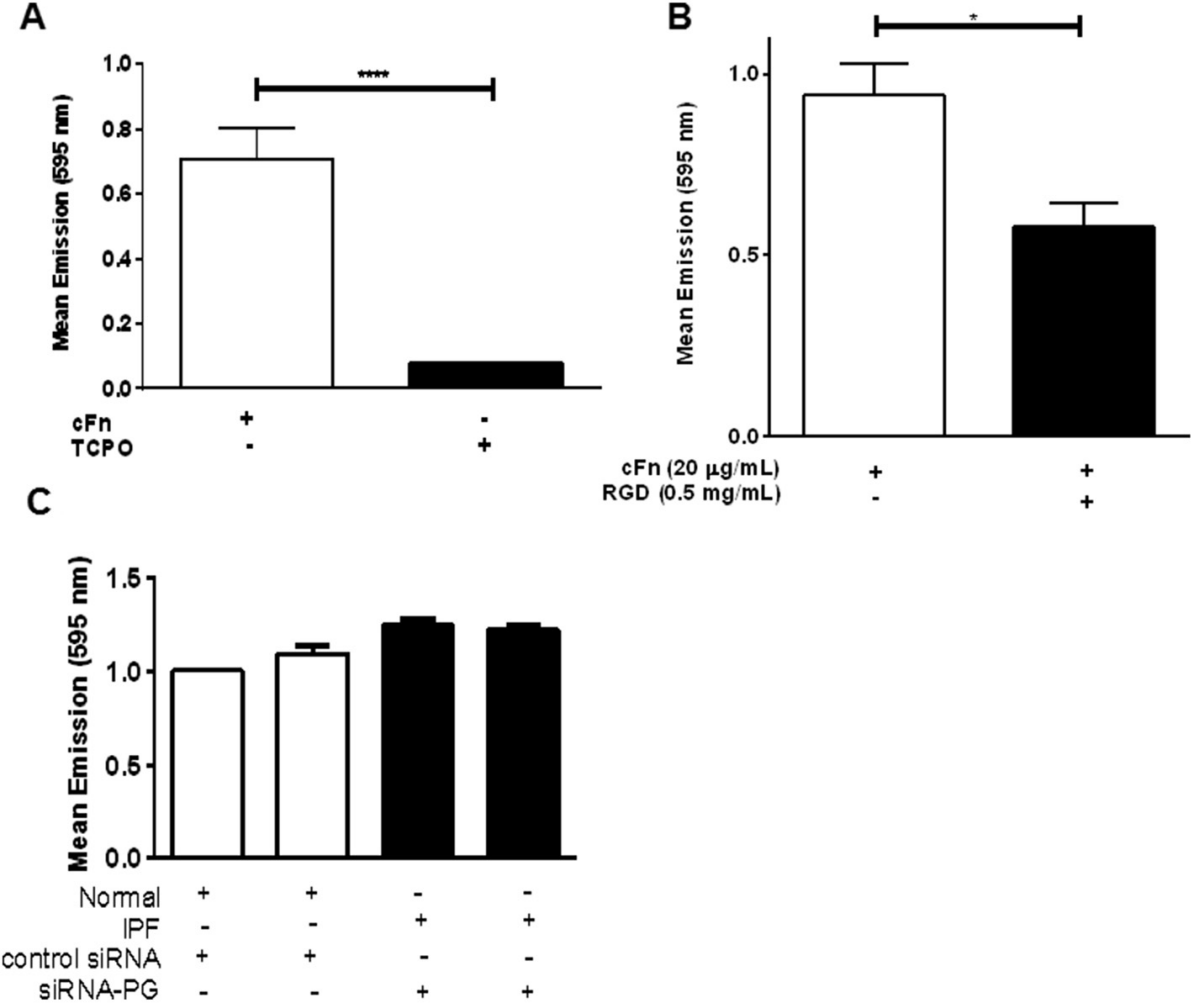
PG expression does not significantly alter cellular adhesion to fibronectin. Cell adhesion assays using cellular fibronectin (cFn) or tissue culture plastic (TCPO) served as a substrate for normal fibroblasts. The cells seeded on cFn had a 6-fold increase in adherence compared to TCPO (*p* < 0.0001 ) (**a**). To ensure cFN has been serving as a proper adhesion substrate, untreated normal cells were treated with an RGD peptide to block the adhesive properties of cFn. Those cells treated with the peptide had 4.5 fold less adhesion compared to the cFN only cells (*p* < 0.02) (**b**). Normal (*n* = 17) or IPF cells (*n* = 14) transfected with either Φ-siRNA or siRNA-PG and placed on the cFn substrate showed no difference in adhesive capacity to fibronectin (**c**)

In pulmonary fibrosis, fibroblasts and myofibroblasts accumulate in fibronectin- and collagen-rich structures termed fibroblastic foci [19]. Since plakoglobin plays a role in cell-cell adhesion, we sought to determine whether we could also identify such a role for plakoglobin in cell-matrix adhesion. Figure 4c demonstrates that there was no significant difference in normal or IPF fibroblasts that were transfected with siRNA-PG or with a control siRNA, Φ-siRNA and seeded on a fibronectin-coated dish (Normal Φ-siRNA and siRNA-PG, *n* = 17 and IPF Φ-siRNA and siRNA-PG, *n* = 14). Notably, there was no significant difference in adhesion between normal and IPF fibroblasts. These data suggest that cell-fibronectin adhesion is independent of plakoglobin. To demonstrate sufficient cell adhesion untreated normal fibroblasts were seeded on cellular fibronectin coated tissue culture plastic or tissue culture plastic only (TCPO)(Fig. 4a). As expected, cells adhered robustly to cellular fibronectin coated wells compared to the TCPO condition. To further validate the efficacy of our adhesion experiments, cellular fibronectin coated wells were treated with a RGD peptide known for blocking the RGD sequence responsible for adhesion in cellular fibronectin [25, 26]. As expected the RGD peptide blocked fibroblast adhesion to cellular fibronectin (Fig. 4b).

**Fig. 5:**
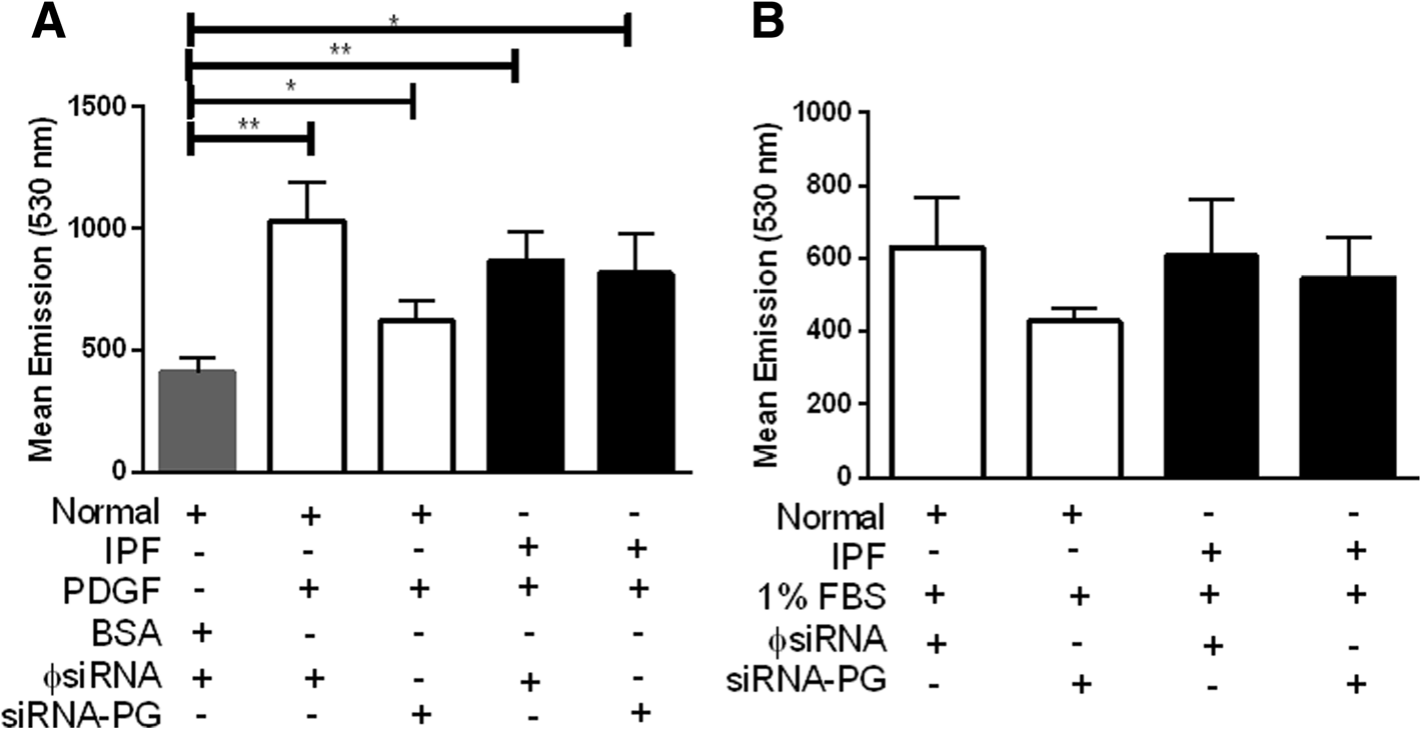
PG expression does not affect proliferation in normal or IPF fibroblasts. The rate of proliferation remained unchanged in both normal and IPF fibroblasts following PG knockdown. Normal cells transfected with ɸsiRNA were cultured in BSA ( *n* = 16) and had a significant reduction in proliferation compared to all other experimental groups stimulated with PDGF which include normal Φ-siRNA (*n* = 6, *p* < 0.01), normal siRNA-PG (*n* = 6, *p* < 0.05), IPF Φ-siRNA (*n* = 6, *p* < 0.002) and IPF siRNA-PG (*n* = 6, *p* < 0.03) (**a**). Both normal and IPF cells treated with either ɸsiRNA or siRNA-PG were also kept in 1 % FBS (**b**) as a control. There were no significant differences in proliferation observed with the PDGF or 1 % FBS treatments groups. Notably, IPF lung fibroblasts were observed to proliferate to a similar degree as normal cells

To assess whether plakoglobin influences fibroblast proliferation, we next seeded cells in a FN-coated 96-well dish. Cells were then transfected with siRNA constructs and serum-starved for 48 hours, followed by a 24-hour incubation in 1 % serum-containing media with or without stimulation by PDGF (Fig. 5a) or TGF-β (data not shown). A BSA control was used on normal control transfected fibroblasts to ensure proper induction of proliferation following transfection. The BSA control group (*n* = 16) had a significantly lower rate of proliferation compared to all of the PDGF-treated experimental groups. These include the normal ΦsiRNA (*n* = 6, *p* < 0.01), normal siRNA-PG (*n* = 6, *p* < 0.05), IPF Φ-siRNA (*n* = 6, *p* < 0.002) and IPF siRNA-PG (*n* = 6, *p* < 0.03) (Fig. 5a). A baseline of proliferation was obtained in Fig. 5b using 1 % serum-containing only media without the stimulation of PDGF or TGF-β. Silencing plakoglobin had no significant impact on the proliferation of either normal or IPF fibroblasts stimulated with serum or PDGF. Although IPF fibroblasts treated with siRNA-PG had a slight decrease in proliferation, there were no significant differences between PG-silenced normal or IPF fibroblasts.

**Fig. 6:**
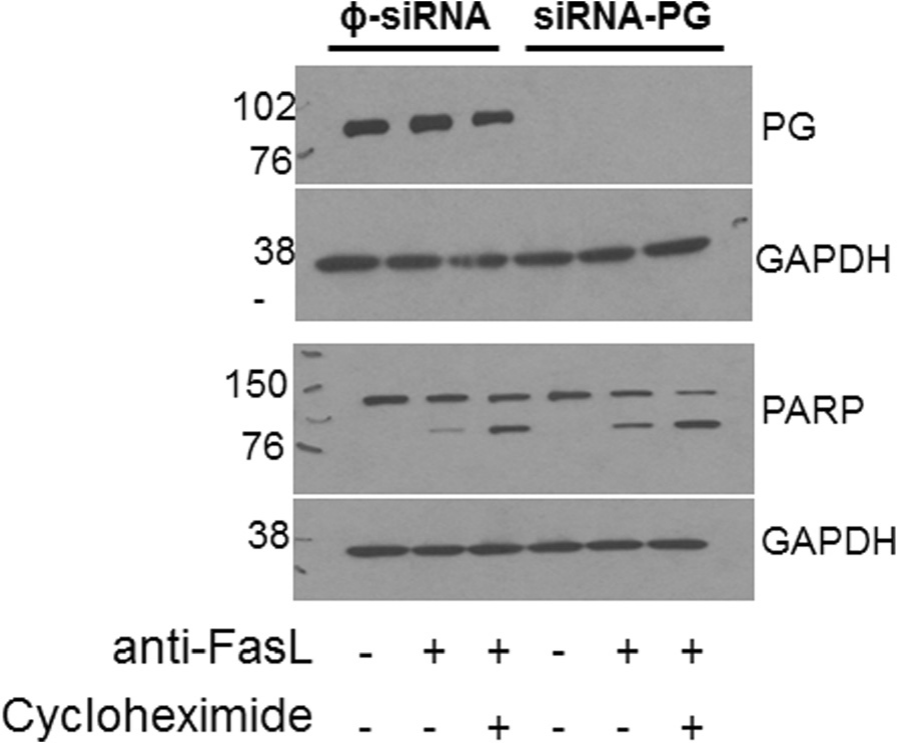
PARP expression analysis in plakoglobin knockdown in IPF fibroblasts. Representative western blots that were successfully silenced for plakoglobin (upper panel, right 3 lanes) compared to the control treated cells (left 3 lanes). Cleaved PARP (116 kDa and 89 kDa) (lower panel) in IPF fibroblasts was assessed in cells treated with (+) or without (−) anti-FasL/Cyclo. GAPDH was used as a loading control

**Fig. 7:**
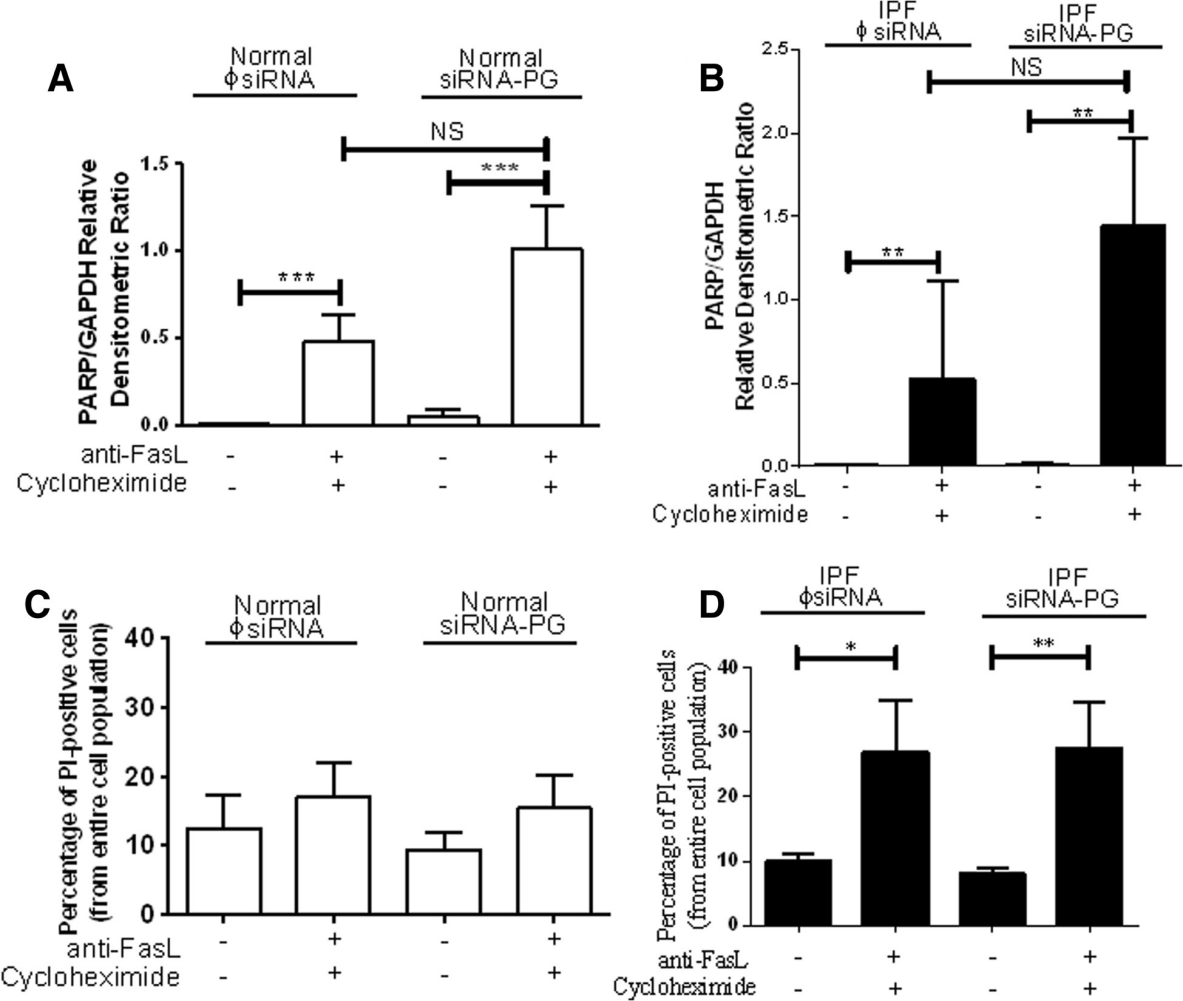
Cleaved PARP protein expression and cell viability in normal and IPF fibroblasts. Transfected normal (**a, c**) and IPF (**b, d**) fibroblasts were treated with (+) or without (−) anti-FasL and cycloheximide for 6 hours (A and B). Results indicate that at 6 hours, the normal (Φ-siRNA (−/−), *n* = 13, Φ-siRNA (+/+), *n* = 16, siRNA-PG (−/−), *n* = 15 and siRNA-PG (+/+), *n* = 12) (**a**) and IPF (Φ-siRNA (−/−), *n* = 8, Φ-siRNA (+/+), *n* = 7, siRNA-PG (−/−), *n* = 7 and siRNA-PG (+/+), *n* = 4) (**b**) fibroblasts transfected with ɸsiRNA or siRNA-PG and treated (+/+) had significantly higher levels of cleaved PARP compared to the transfected cells not treated (−/−) (*p* < 0.0003, *p* < 0.0002, *p* < 0.003 and *p* < 0.01, respectively). (**a**). There was no significance found between the ɸsiRNA and siRNA-PG experimental groups in either normal or IPF fibroblasts. PI levels were assessed by flow cytometry after overnight treatment with (+) or without (−) anti-FasL/cycloheximide in normal and IPF fibroblasts (c and d). The normal transfected fibroblasts did not show any significant change in PI positivity between the treated (+/+) and untreated (−/−) transfected cells (Φ-siRNA (−/−), *n* = 7, Φ-siRNA (+/+), *n* = 8, siRNA-PG (−/−), *n* = 6 and siRNA-PG (+/+), *n* = 8) (**c**). The IPF fibroblasts did show a significant difference between the treated (+/+) and untreated (−/−) transfection groups (ɸsiRNA or siRNA-PG, *p* < 0.05 and *p* < 0.005, respectively). However, there is a lack of significance between the ɸsiRNA and siRNA-PG groups treated with anti-FasL/Cyclo (+/+) (Φ-siRNA (−/−), *n* = 7, Φ-siRNA (+/+), *n* = 7, siRNA-PG (−/−), *n* = 7 and siRNA-PG (+/+), *n* = 7) (**d**)

One hallmark of IPF is the presence of relatively apoptosis-resistant fibroblasts [27, 28]. Since prior data suggests a role for plakoglobin in mediating apoptosis [29, 30], we next transfected primary normal or IPF fibroblasts with siRNA-PG or ΦsiRNA constructs followed by induction of apoptosis using a Fas-activating antibody and cycloheximide for 6 hours (*N* = 16 for both normal and IPF cells transfected with either ΦsiRNA or siRNA-PG) [23, 27, 31]. A representative western blot of IPF fibroblasts that have been successfully silenced and probed for cleaved PARP expression is shown in Fig. 6. The normal fibroblasts were treated and assessed in the same manner (data not shown). As predicted, and consistent with Fig. 6, we observed a significant induction of apoptosis in cells treated with the combination of anti-FasL and Cycloheximide (+/+), as indicated by the level of cleaved PARP (89/116 kDa) expression relative to GAPDH after 6 hours of treatment (Fig. 7 panels a and b, normal ΦsiRNA (+/+) vs. normal ΦsiRNA (−/−) *p* < 0.0003, normal siRNA-PG (+/+) vs. normal siRNA-PG (−/−), *p* < 0.0002, IPF ΦsiRNA (+/+) vs. IPF ΦsiRNA (−/−) and IPF siRNA, *p* < 0.01, respectively). Notably, silencing plakoglobin did not appear to enhance apoptosis in IPF fibroblasts treated with the combination of Fas-activating antibody and cycloheximide (Fig. 7 panel b, *p* <0.01). To assess cell viability in normal and IPF fibroblasts after 6 hours of treatment with or without siRNA-PG and anti-FasL/Cycloheximide, cells were stained for propidium iodide (PI) and analyzed through flow cytometry. Silencing PG led to no significant difference in cell death in normal (7C) or IPF (7D) fibroblasts as indicated by PI staining (Fig. 7, panels c and d).

## Discussion

Due to both the importance of normal plakoglobin expression and the high level of homology between plakoglobin and β-catenin, there have been great efforts towards characterizing the role that plakoglobin may play in the Wnt/β-catenin pathway. Our data suggest that critical fibroblast functions in IPF are not regulated by the presence of plakoglobin.

A challenge in studying IPF is the heterogeneity of the disease across a wide spectrum of patients [32–34]. This heterogeneity results in seemingly non-significant trends in biochemical analysis across several samples as exemplified in the plakoglobin protein expression profiles shown in Fig. 2. Despite this difference there is still a significant reduction in overall plakoglobin protein detected in IPF samples versus normal fibroblasts. The RNA levels between the normal and IPF lung tissue remains non-significant, which suggests that plakoglobin may undergo alternative splicing and/or other post-translational modifications. Since other studies have shown that a reduction in plakoglobin leads to aberrant cellular processes including adhesion [35], proliferation [36] and apoptosis [37], we examined these physiological processes in normal and IPF lung fibroblasts.

Cell-matrix interactions are thought to drive fibroblast phenotypic behaviors in IPF [21, 38]. Thus, we hypothesized that silencing plakoglobin might affect cellular adhesion to fibronectin, a major protein found in fibroblastic foci in IPF [20, 39]. Even though we did not identify a significant difference in matrix adhesion before or after plakoglobin silencing (Fig. 4c), we cannot exclude the possibility that other, non-fibronectin-binding integrin subunits might have been affected by plakoglobin silencing.

Since IPF is a fibroproliferative disease, and the level of proliferative capacity of activated fibroblasts may be increased in IPF patients [40], we sought to determine whether abnormal plakoglobin expression altered the proliferative response to PDGF or TGF-β in normal and IPF fibroblasts. While we did not observe an effect of plakoglobin silencing on fibroblast proliferation, our data appear to be in agreement with a second study showing no difference in proliferation in plakoglobin-null keratinocytes [29]. Together these data seem to suggest that neither baseline nor PDGF-stimulated proliferation is dependent on plakoglobin. The lack of response to PDGF in the IPF fibroblasts is consistent with several prior reports [41–43]. A study on IPF fibroblast proliferation has made it clear that the location and age of the IPF fibroblasts will affect the proliferative rate of the cells [41]. Usual interstitial pneumonia, the histologic hallmark of IPF, is a highly heterogeneous injury pattern where highly fibrotic areas can be adjacent to normal tissue. Thus, sampling lung fibroblasts using a standard explant method may account for variations in fibroblast behavior seen in our study.

One of the hallmarks of IPF is an overabundance of myofibroblasts localized to areas of active matrix synthesis termed fibroblastic foci. Staining fibroblastic foci in IPF tissue revealed that the epithelium and not the myofibroblasts express apoptotic markers [40]. This is supported by other studies that suggest IPF myofibroblasts are more resistant to Fas-induced apoptosis [31, 44, 45]. Research findings propose that plakoglobin plays a role in regulating cellular survival. Two separate studies using keratinocytes with a reduced level of plakoglobin demonstrated both a decrease in cell-cell contact and a reduction in apoptosis [29, 46]. Other studies suggest that plakoglobin increases the likelihood of a cell to undergo apoptosis, whereas without endogenous plakoglobin the cells are unable to release cytochrome c from the mitochondria and therefore are deficient in activating caspase-3 in response to apoptotic stimuli [29, 30]. Thus, we hypothesized that decreased plakoglobin expression seen in IPF fibroblasts might account for the relative resistance to apoptosis. On the contrary, our results did not indicate any significant difference in the expression of apoptotic markers cleaved PARP (Figs. 6 and 7, panels a and b) or propidium iodide (Fig. 7, panels c and d) between normal and IPF fibroblasts when PG was silenced with siRNA. However, it is possible that cells with low plakoglobin are resistant to apoptosis from other stimuli; this hypothesis will require further testing.

Our knockdown efficiency for plakoglobin was approximately 80 % (Fig. 3). There may be more robust differences in phenotypic behavior of fibroblasts that have PG protein expression completely eradicated. Further studies using a construct to effectively knockout PG would be the next series of experiments to pursue. Despite these results, the down regulation of plakoglobin in IPF lung fibroblasts may well have other, as yet unidentified, effects on cellular behavior and/or disease progression. Our data suggest that further study into the role of plakoglobin in IPF is warranted to identify a functional consequence of the observed decreased protein expression.

## Conclusions

In summary, previous studies suggest that plakoglobin affects cell-cell and cell-matrix interactions in various systems. However, our data indicate that plakoglobin may not have a similar effect in healthy or diseased lung fibroblasts.

## Abbreviations

AJ: Adherens junction
β-Catenin: Beta-catenin
Cyclo: Cycloheximide
DAPI 4′: 6-diamidino-2-phenylindole
DMEM: Dulbecco’s modified eagle’s medium
Fn: Fibronectin
GAPDH: Glyceraldehyde 3-phosphate dehydrogenase
PARP: Poly ADP ribose polymerase
PG: Plakoglobin
PI: Propidium iodide
φsiRNA-PG: Control PG silencing construct
TCPO: Tissue culture plastic only
